# Increased endothelin-1 in colorectal cancer and reduction of tumour growth by ET _A_ receptor antagonism

**DOI:** 10.1054/bjoc.2001.2193

**Published:** 2001-11

**Authors:** E Asham, A Shankar, M Loizidou, S Fredericks, K Miller, P B Boulos, G Burnstock, I Taylor

**Affiliations:** Departments of Surgery, Histopathology, The Royal Free and University College Medical School, 67–73 Riding House Street, London, W1W 7EJ; The Autonomic Neuroscience Institute, The Royal Free and University College Medical School, Rowland Hill Street, London, NW3 2PF; The Analytical Unit, St George's Hospital Medical School, Blackshaw Road, Tooting, London, SW17 0QT, UK

**Keywords:** ET-1, colorectal cancer, colorectal liver metastases, ET _A_ antagonist, ET _B_ antagonist, BQ123

## Abstract

Endothelin-1 (ET-1) is a vasoconstrictor peptide which stimulates proliferation in vitro in different cell types, including colorectal cancer cells. Raised ET-1 levels have been detected both on tissue specimens and in the plasma of patients with cancers. To investigate the role of ET-1 in colorectal cancer: (i) ET-1 plasma levels in patients with colorectal cancer were measured by radioimmunoassay: group 1 = controls (*n* = 22), group 2 = primary colorectal cancer only (*n* = 39), group 3 = liver metastases only (*n* = 26); (ii) ET-1 expression in primary colorectal cancer specimens (*n* =10) was determined immunohistochemically and (iii) the effect of intraportally infused antagonists to the two ET-1 receptors, ET _A_ and ET _B_, on the growth of liver metastases in a rat model was assessed. ET-1 plasma levels were significantly increased in both patients with primary tumour and patients with metastases, compared to controls (*P* < 0.01, 3.9 ± 1.4, 4.5 ± 1.5, vs. 2.75 ± 1.37 pg/ml, respectively). Immunohistochemically, strong expression of ET-1 was found in the cytoplasm, stroma and blood vessels of cancers, unlike the normal colon where only the apical layer of the epithelium, vascular endothelial cells and surrounding stroma were positively stained. In the rat model, there was significant reduction in liver tumour weights compared to controls, following treatment with the ET _A_ antagonist (BQ123) 30 min after the intraportal inoculation of tumour cells (*P* < 0.05). These results suggest ET-1 is produced by colorectal cancers and may play a role in the growth of colorectal cancer acting through ET _A_ receptors. ET _A_ antagonists are indicated as potential anti-cancer agents. © 2001 Cancer Research Campaign http://www.bjcancer.com


*British Journal of Cancer* (2001) **85**(11), 1759–1763
© 2001 Cancer Research Campaign
doi: 10.1054/ bjoc.2001.2193, available online at http://www.idealibrary.com on IDEAL

http://www.bjcancer.com

### Increased endothelin-1 in colorectal cancer and reduction of tumour growth by $ET_A$ receptor antagonism

**E Asham, A Shankar, M Loizidou, S Fredericks[3], K Miller[1], PB Boulos, G Burnstock[2] and I Taylor**

Departments of Surgery and [1]Histopathology, The Royal Free and University College Medical School, 67–73 Riding House Street, London W1W 7EJ; [2]The Autonomic Neuroscience Institute, The Royal Free and University College Medical School, Rowland Hill Street, London NW3 2PF; [3]The Analytical Unit, St George's Hospital Medical School, Blackshaw Road, Tooting, London SW17 0QT, UK

**Summary** Endothelin-1 (ET-1) is a vasoconstrictor peptide which stimulates proliferation in vitro in different cell types, including colorectal cancer cells. Raised ET-1 levels have been detected both on tissue specimens and in the plasma of patients with cancers. To investigate the role of ET-1 in colorectal cancer: (i) ET-1 plasma levels in patients with colorectal cancer were measured by radioimmunoassay: group 1 = controls ($n$ = 22), group 2 = primary colorectal cancer only ($n$ = 39), group 3 = liver metastases only ($n$ = 26); (ii) ET-1 expression in primary colorectal cancer specimens ($n$ =10) was determined immunohistochemically and (iii) the effect of intraportally infused antagonists to the two ET-1 receptors, $ET_A$ and $ET_B$, on the growth of liver metastases in a rat model was assessed. ET-1 plasma levels were significantly increased in both patients with primary tumour and patients with metastases, compared to controls ($P < 0.01$, 3.9 ± 1.4, 4.5 ± 1.5, vs. 2.75 ± 1.37 pg/ml, respectively). Immunohistochemically, strong expression of ET-1 was found in the cytoplasm, stroma and blood vessels of cancers, unlike the normal colon where only the apical layer of the epithelium, vascular endothelial cells and surrounding stroma were positively stained. In the rat model, there was significant reduction in liver tumour weights compared to controls, following treatment with the $ET_A$ antagonist (BQ123) 30 min after the intraportal inoculation of tumour cells ($P < 0.05$). These results suggest ET-1 is produced by colorectal cancers and may play a role in the growth of colorectal cancer acting through $ET_A$ receptors. $ET_A$ antagonists are indicated as potential anti-cancer agents. © 2001 Cancer Research Campaign    http://www.bjcancer.com

**Keywords**: ET-1; colorectal cancer; colorectal liver metastases; $ET_A$ antagonist; $ET_B$ antagonist; BQ123

Endothelin-1 (ET-1), a recognised vasoconstrictor peptide, has been implicated in the growth regulation of several tumours. Its actions are mediated via two receptor subtypes $ET_A$ and $ET_B$ (Yanagisawa et al, 1988; Clozel and Clozel 1989; Clozel et al, 1992). Both receptors have a role in vasomotor control, although only $ET_A$ appears to be involved in the mitogenic action of ET-1 (Ali et al, 2000a; Bagnato et al, 1997, 1999; Nelson et al, 1996; Kitigawa et al, 1994).

ET-1 stimulates proliferation in vitro of fibroblasts, renal mesangial cells, smooth muscle and several tumour cell lines, including colorectal cancer (Hirata et al, 1989; Kusuhara et al, 1989; Simonson et al, 1989; Shichiri et al, 1991; Ali et al, 2000a). Immunohistochemical studies on breast and prostate cancer demonstrate increased expression of ET-1, with patients exhibiting elevated plasma levels of ET-1 (Kojima and Nihei, 1995; Nelson et al, 1995). Other malignant tumours associated with increased ET-1 plasma levels include pancreatic and hepatocellular carcinomas (Oikawa et al, 1994; Nakamuta et al, 1993).

We have previously shown in a pilot study that patients with colorectal cancer, with and without liver metastases, have elevated plasma levels of ET-1 and that the peptide is overexpressed by different cell types within colorectal liver metastases (Shankar et al, 1998). ET-1 binding sites in primary colorectal cancer are known to be unregulated (Inigagi et al, 1992). Recently, we investigated binding to receptor subtypes and showed specifically that $ET_A$ receptors are overexpressed while $ET_B$ receptors are underexpressed in colorectal cancer tissue compared to normal colon (Ali et al, 2000b). These results suggest ET-1 may play a paracrine or autocrine role in the development of colorectal cancer both locally and systemically.

The purpose of this study is to explore the role of ET-1 in colorectal cancer from three perspectives: firstly, to confirm in a further series of observations the elevated plasma levels of ET-1 in patients with colorectal cancer; secondly, to assess the expression of ET-1 in primary colorectal cancer specimens using immunohistochemistry; and finally to assess the effect of intraportally infused $ET_A$ and $ET_B$ receptor antagonists on the growth of liver metastases in an animal model.

### METHODS

### Assay for circulating ET-1

#### *Blood collection*

Three groups of patients were studied: Group 1—controls ($n$ = 22), group 2—patients with primary colorectal cancer without liver metastases on CT scanning or at laparotomy ($n$ = 39), group 3—patients with liver metastases whose primary colorectal cancer had already been resected ($n$ = 26). Information was collected on serum carcinoembryonic antigen (CEA) levels and Dukes stage, for all patients.

Patients with the following conditions—which are independently associated with elevated ET-1 levels—were excluded;

*Received 11 September 2001*
*Accepted 20 September 2001*

*Correspondence to:* M Loizidou hypertension, renal failure, heart disease, pre-existing liver disease and Raynauds phenomenon. We have previously demonstrated that elevated ET-1 plasma levels are not associated with endothelial cell trauma (as determined by the plasma levels of thrombomodulin, a marker of endothelial cell integrity) and as such represent an increased production of ET-1 rather than a consequence of tumour invasion and damage of adjacent blood vessels.

All patients had a fasting venous specimen taken prior to surgery. This was collected into precooled blood bottles containing ethylene diamine tetraacetic acid. The specimens were then centrifuged in a chillspin (4˚ for 15 min at 1000 ***g***) and the plasma frozen at –70˚ within 1 h of collection.

### *ET-1 radioimmunoassay*

Plasma ET-1 was measured by radioimmunoassay (RIA, Nichols Institute Diagnostics Ltd, Saffron Walden, Essex, UK) according to the manufacturer's instructions. The anti ET-1 antibody had a cross-reactivity of 67% with ET-2, 84% with ET-3, 2.6% with Big ET-1, 5.3% with Big ET-2 and 0.2% with Big ET-3. Endothelin concentrations were calculated by plotting the % bound against log concentrations of known standards.

### Immunohistochemistry for ET-1

Specimens of primary colorectal cancer and normal colonic tissue (taken 10 cm away from the tumour) were collected at the time of resection from 10 patients, fixed in neutral buffered formalin for 24 h and paraffin wax embedded.

Sections were cut (5 µm thick), dewaxed in xylene, rehydrated through decreasing concentrations of ethanol and washed in Tris Buffered Saline (TBS, 3 × 5 min). Sections were taken to water and antigen unmasking was achieved by microwaving slides in 600 ml of citrate buffer (0.01 M, pH = 6), for 20 min (full power, 800 W). Hot buffer was flushed out with cold running water and sections were transferred to the incubation chamber and washed with TBS. The protocol for ET-1 staining was as follows: Tissue endogenous peroxidase was blocked by incubating the sections for 10 min in 0.5% methanolic $H_2O_2$. Sections were washed in running tap water and incubated in mouse monoclonal anti-ET-1 antibody for 1 h (1:100 in TBS, Department of Immunology, University College London, UK, Ong et al, 1993; the antibody cross-reacts with ET-2 and ET-3 (15%), but not big ET). The specimens were washed again in TBS and incubated with rabbit anti-mouse immunoglobulin for 35 min. Routine streptavidin-biotin peroxidase complexing followed (Dako Ltd, Ely, UK) according to the manufacturer's instructions. For the final colour reaction, freshly prepared 3,3 diaminobenzidine (DAB) solution incorporating 0.03% $H_2O_2$ was applied on the slides (10 min). DAB was rinsed off with TBS and sections were washed in running tap water. Nuclei were counterstained with haematoxylin (4 min), dehydrated and mounted.

### Assessment of ET-1 antagonists in an animal model of colorectal liver metastases

### *In vivo protocol*

The animal model used for colorectal liver metastases has been described in detail previously (Loizidou et al, 1991). The intention of this component of the study was to assess the effect of intraportally inoculated antagonists to $ET_A$ and $ET_B$ on the development of liver metastases when given at varying times after tumour inoculation. Given the vasoconstricting effect of ET-1 primarily mediated via $ET_A$, application of $ET_A$ and mixed $ET_A$/$ET_B$ antagonists lead to vasodilation and subsequent hypotension, while application of the $ET_B$ antagonist lead to hypertension; as such the doses of antagonists used in all arms of the study were those which produced an approximate 20% change in systemic blood pressure (confirmed in a small series of experiments in our lab). This level of blood pressure change was arbitrarily chosen as the safety limit. The study was contacted under a Home Office approved project licence.

Male Hooded Lister rats weighing between 250–300 ***g*** were anaesthetised using halothane inhalation. A lower midline incision was then made and a tributary of the ileocolic vein was cannulated. All animals received a bolus injection of $1 \times 10^6$ MC28 syngeneic tumour cells in 0.2 ml saline (the dose known to produce reproducible tumour growth by 14 days, Loizidou et al, 1991).

In the first arm of the study the effect of infusions of ET-1 antagonists 30 min after tumour inoculation were assessed. The animals were divided into 5 groups (6 animals per group) and received; BQ123 (Alexis Corporation, Nottingham, UK) an $ET_A$ antagonist, PD142893 (Alexis Corporation) a nonselective $ET_A$ and $ET_B$ antagonist, A-192621 an $ET_B$ receptor antagonist (kindly donated by Abbott, Chicago, II, USA), saline only and saline plus an intramuscular injection of 0.1 mg/kg acepromazine, (National Veterinary Supplies, Stoke-on-Trent, UK) a hypotensive agent which acts as a control for the vascular effects of ET-1 antagonism. Each antagonist was infused at a rate of 100 µg/kg/min for 30 min.

In the second arm of the study, the ileocolic vein was cannulated after tumour cell inoculation was performed. A fine 2F gauge portacath (Becton-Dickinson UK Ltd, Oxford, UK) was tunnelled under the skin to the interscapular area. This allowed access to the portal circulation without the need for a further laparotomy. The animals were then divided into 4 groups (6 animals per group) with infusions of agents given via the portal circulation on days 2, 4 and 6 after tumour innoculation. The infusions were administered under halothane anaesthesia for 30 min, at the same rate as in the previous study. The groups were; saline, BQ123, PD142893, A-192621 and saline plus intramuscular acepromazine.

Given that different passages of the same tumour produce different degrees of percentage hepatic replacement with tumour, in order to compare the different agents one animal from each group received tumour from the same passage. This allowed comparisons to be made between the animals in each of the groups and was applied to both arms of the study.

All animals were sacrificed 14 days after intraportal tumour inoculation, the livers were harvested and the tumours dissected out. Wet weights were obtained for uninvolved liver and for total tumour, per animal. The percent hepatic replacement (PHR) with tumour was calculated for every animal, by dividing tumour weight by total liver (i.e., liver plus tumour) weight and then multiplying by 100. Results were presented as ratios between PHRs of experimental animals to PHRs of control animals.

### RESULTS

### Plasma levels of ET-1

The plasma levels of ET-1 in the control group (age range 42–82 years, median age 59.5 years, 8 females and 14 males, $n = 22$) had a mean of 2.75 pg/ml, (SD = 1.37). In the colorectal cancer group

 *© 2001 Cancer Research Campaign*

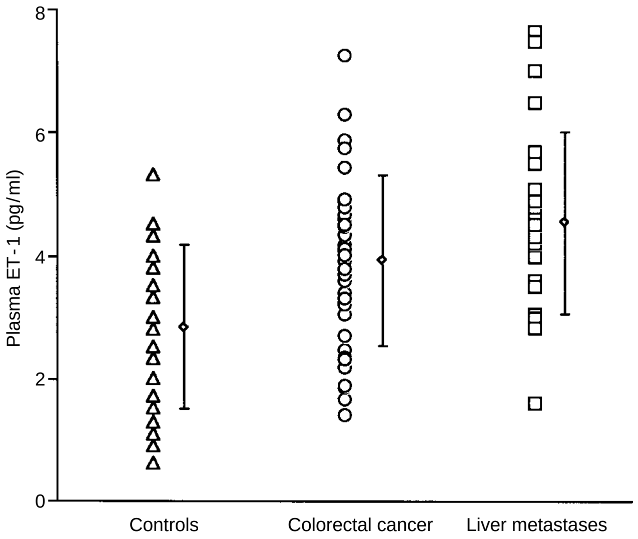

**Figure 1** Levels of ET-1 in plasma from patients with or without colorectal cancer. Individual values are shown for controls (△, *n* = 22), patients with primary colorectal cancer (○, *n* = 39), and patients with previously removed primary colorectal cancer who have subsequently developed liver metastases (□, *n* = 26). Bars are means ( ± SD). Significance was reached with both patient groups compared to controls (*P* < 0.01, Mann–Whitney test)

without metastases (median age 64.6 years, range 55–77 years, 16 females and 23 males, *n* = 39) the mean ET-1 level was 3.9 pg/ml, (SD = 1.4) and in the group of patients with liver metastases (median age 55, range 46–75 years, 3 females and 23 males, *n* = 26) the mean ET-1 level was 4.5 pg/ml (SD = 1.5), as illustrated in Figure 1. There was a significant difference between the cancer patients and the control group (*P* < 0.001 and *P* < 0.01 for the metastases and primary cancer groups, respectively) whilst there was no significant difference between the two groups of cancer patients.

In the colorectal cancer group without metastases, there were 3 Dukes A, 26 Dukes B, and 10 Dukes C patients with CEA levels ranging from <1–43 (median = 2). All 26 patients with liver metastases were classed as Dukes D, by definition, with CEA levels recorded between <1–1650 (median = 11). No correlation was found between ET-1 levels and serum CEA or Dukes stage.

### ET-1 Immunohistochemistry

Five patients had Dukes B and 5 had Dukes C carcinomas (3 females and 7 males). ET-1 immunohistochemistry of control tissue showed ET-1 present in the apical layer of colonic epithelium in 5 of 10 patients studied, vascular endothelial cells and surrounding stroma (Figure 2A). In the colorectal cancer specimens there was strong expression of ET-1 in the cytoplasm of epithelial cells, stroma and blood vessels, in all the tumour specimens studied (Figure 2B). There was no correlation between Dukes staging and intensity of staining.

### ET-1 antagonists

### *Day 0 infusion group*

The only group which demonstrated a significant reduction in tumour load compared to the saline control group (paired *t*-test) was those animals which received BQ123, The mean ratio (compared to the saline control group) was 0.64 ± 0.20, (*P* < 0.05, paired *t*-test).

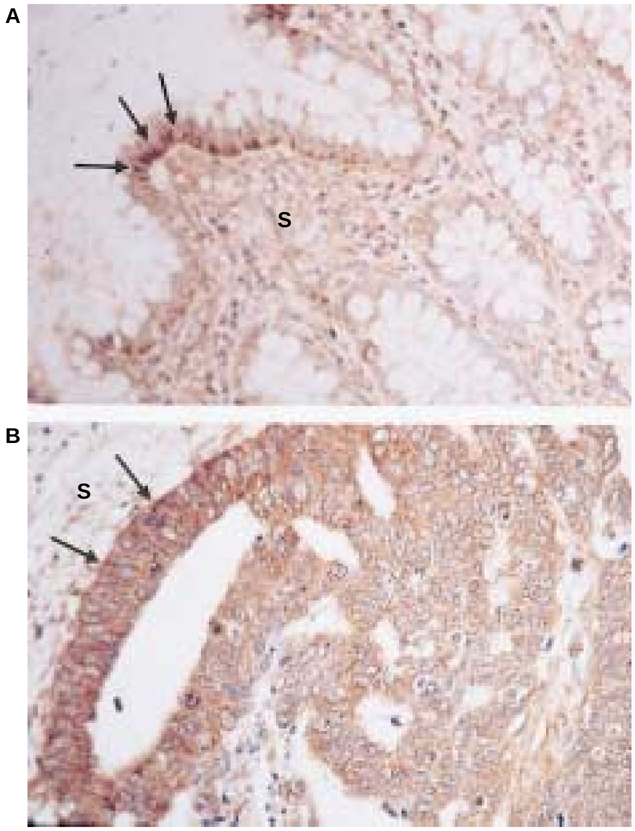

**Figure 2** ET-1 immunoreactivity in colon. (**A**) Control: note ET-1 staining (brown) limited to the apical segment of the colonic epithelium (arrows) together with some staining of the stroma (S). (**B**) Colorectal cancer showing strong staining for ET-1, in the cytoplasm of epithelial cancer cells (arrows) and in the stroma (S). Counstertained with haematoxylin. Magnification × 700

PD142893 gave a mean of 1.10 ± 0.9 (NS), A-192621 was 1.13 ± 0.88 (NS) and the saline/acepromazine group was 1.4 ± 0.70 (NS). These results are illustrated in Figure 3, which demonstrates reductions in tumour load compared to the saline only group.

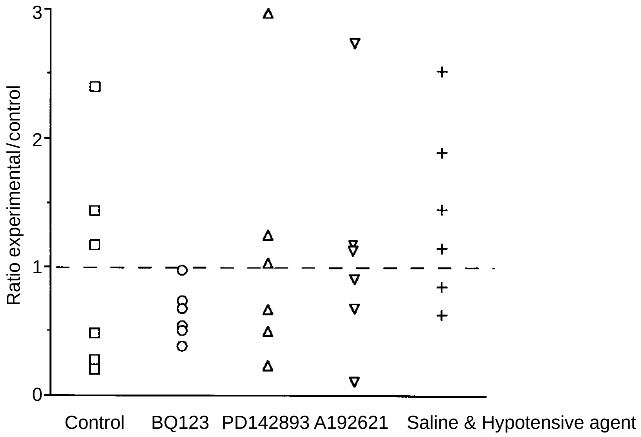

**Figure 3** Liver tumours in rats which received Endothelin receptor antagonists. After tumour cell inoculation, animals received one of the following: saline (controls □, dotted line), BQ123 (○), PD142893 (△), A-192621 (▽), or saline plus the hypotensive agent acepromazine (+) (*n* = 6 per group). Fourteen days later, liver tissue (tumour and normal) was weighed. Results (Y-axis) are shown as a ratio of the % hepatic replacement by tumour (PHR) in the experimental animals compared to control PHR. The horizontal dotted line represents the average (mean) of control values (SD = 0.23). The BQ123 group had significantly smaller tumours than the controls (*P* < 0.05, paired Student *t*-test)

### *Day 2, 4 and 6 group*

In this group of experiments, none of the agents produced significant reductions in tumour load compared to the control group. The mean values (SD) were 1.11 ± 0.81 for BQ123, 1.02 ± 0.58 for PD142893, and 1.80 ± 1.90 for the saline/acepromazine group.

### DISCUSSION

ET-1 is a vasoconstrictor peptide the physiological functions of which are well described. ET-1 acts via two G-linked protein receptors $ET_A$ and $ET_B$, with $ET_A$ mediating vasoconstriction and $ET_B$ vasodilation (Yanasigawa et al, 1988; Clozel and Clozel, 1989; Clozel et al, 1992).

Recently ET-1 has been implicated as a possible regulator of tumour growth in a number of human malignancies including colorectal cancer (Bagnato et al, 1997, 1999; Kojima and Nihei, 1995; Nelson et al, 1995, 1996, 1999; Oikawa et al, 1994; Nakamuta et al, 1993; Shankar et al, 1998; Ali et al, 2000a). A variety of tumour cell lines including colorectal cancer produce ET-1, which when added to the growth medium results in an increase in tumour cell turnover (Shichiri et al, 1991; Bagnato et al, 1997; Moraitis et al, 1977). Studies involving colorectal, ovarian and prostate cancer suggest that the receptor responsible for the ET-1 mitogenic action is $ET_A$, which is probably upregulated (Bagnato et al, 1997, 1999; Nelson et al, 1995; Ali et al, 2000a). Binding of ligand to receptor usually activates phospholipase C followed by $Ca^{++}$/protein kinase C signalling. It has also been shown that ET-1 acts as a co-mitogen with other factors such as epidermal growth factor, EGF (Bagnato et al, 1997). Synergism might be in part explained by the fact that $Ca^{++}$/protein kinase C cross-talk with (activate) molecules belonging to EGF-initiated tyrosine kinase cascades within the cell (Bagnato et al, 1997; Luttrell et al, 1999). In a recent study (Eberl et al, 2000), a mixed $ET_A/ET_B$ receptor antagonist potentiated FasL-induced apoptosis in cultured colorectal cancer cells. This suggests that ET-1 may not only enhance growth, but also protect against apoptosis in certain cell types.

In colorectal cancer Inagaki et al (1992) demonstrated a high density of ET-1 binding sites within tumour vessels and stromal tissues surrounding primary colorectal cancers. Shankar et al (1998) using immunoelectron microscopy have demonstrated increased expression of ET-1 by a variety of cell types within colorectal liver metastases and that patients with colorectal cancer with and without liver metastases had elevated plasma levels of ET-1. Such findings suggest ET-1 may act by a paracrine/autocrine mechnism in these tumours.

This study has extended the earlier work with regard to elevated plasma levels of ET-1 in patients with colorectal cancer (Shankar et al, 1998) and also shows that primary tumours as well as liver metastases produce ET-1. These findings complement recently published data on Big ET-1, the precursor of ET-1, in colorectal cancer (Simpson et al, 2000). The authors found significantly higher plasma levels of Big ET-1 in patients with cancer compared to controls and that tissues from colorectal cancer overexpress Big ET-1, compared to controls. Immunohistochemically, we demonstrated that in colorectal cancer tissue, cancer epithelial cells, endothelial cells and stromal cells overexpress ET-1, compared to normal colon. The fact that, in some cases, normal colonic mucosa also expresses ET-1 suggests it may play a role in the control of normal cell shedding and perhaps apoptosis as suggested by other groups (Shichiri et al, 1998). It has been proposed that primary tumours control the growth of distant metastases by the secretion of a variety of messenger compounds (Hanahan and Folkman, 1998) and ET-1 may have such a role.

During the metastatic cascade, tumour emboli are shed from primary colorectal cancers and reach the liver via the portal circulation. These micrometastases initially obtain their blood supply from the portal vein. This is the basis for the rationale behind perioperative portal vein chemotherapy (Shankar et al, 1996).

Since ET-1 appears to play a role in colorectal cancer development, administration of intraportally infused ET-1 antagonists at this micrometastatic phase might produce a reduction in hepatic tumour load. We have previously shown that MC28 tumours possess similar anatomical and physiological profiles to colorectal liver metastases and also express elevated levels of ET-1 (Loesch et al, 1997; Ashraf et al, 1997). As such, the behaviour of these tumours should mimic those of colorectal cancer.

The findings in this study confirm the potential involvement of $ET_A$ receptors in the development of colorectal liver metastases. Only the $ET_A$ antagonist BQ123 produced a significant reduction in tumour weight. Also of interest is the timing of delivery; since only BQ123 given at the time of inoculation, but not if administered at 2, 4, or 6 days after tumour inoculation, produced any effect. These findings are in keeping with previous studies involving cytotoxic portal vein infusions designed to prevent hepatic tumour development, and suggest the importance of peri-inoculation infusions compared to “delayed” treatment (Sutanto-Ward et al, 1992). One explanation for this finding is that after 24–48 h these tumours may switch their blood supply from the portal vein to the hepatic artery and hence insufficient antagonist reaches the tumour. Another suggestion is that the role of ET-1 in metastatic development is to promote tumour implantation and the initial establishment of micrometastases, Accordingly, the administration of these antagonists after this stage would have little effect. Further experimentation with different antagonists and different in vivo models would be necessary to elucidate possible mechanisms of action. Use of recently reported $ET_A$ antagonists that could be delivered systemically (or orally) may resolve this issue (Liu et al, 1998; Wilson et al, 1999).

The absence of any effect in the hypotensive control group suggests that changes in blood flow caused by ET-1 receptor antagonism are not the cause of the effect seen.

These results suggest that ET-1 is produced by colorectal cancer, and may play a role both locally and systemically in cancer development by acting through $ET_A$ receptors. In addition application of $ET_A$ antagonists via the portal vein at the time of tumour implantation reduces subsequent hepatic involvement in this model of colorectal liver metastases.

### ACKNOWLEDGEMENTS

We are grateful to the Middlesex Hospital Special Trustees, London, W1, for financial support for this project. We thank Abbott Laboratories, Chicago, Illinois for donating endothelin receptor antagonists. We thank Dr R. Jordan and Mr T. Robson for their invaluable help with imaging.

### REFERENCES

Ali H, Loizidou M, Dashwood M, Savage F, Sheard C and Taylor I (2000a) Stimulation of colorectal cancer cell line growth by ET-1 and its inhibition by ETA antagonists. *Gut* **47**: 685–688

 *© 2001 Cancer Research Campaign*

Ali H, Dashwood M, Dawas K, Loizidou M, Savage F and Taylor I (2000b) Endothelin receptor expression in colorectal cancer. *J Cardiovasc Pharm* **36**: S69–S71

Ashraf S, Loizidou M, Crowe R, Turmaine M, Burnstock G and Taylor I (1997) Blood vessels in liver metastases from both sarcoma and carcinoma lack perivascular innervation and smooth muscle cells. *Clin Exp Metastasis* **15**: 484–498

Bagnato A, Tecce R, Dicastro V and Catt KJ (1997) Activation of mitogenic signalling by endothelin-1 in ovarian carcinoma cells. *Cancer Res* **57**: 1306–1311

Bagnato A, Salani D, Di Castro V, Wu-Wong JR, Tecce R, Nicotra MR, Kenuti A and Natali PG (1999) Expression of ET-1 and $ET_A$ receptor in ovarian carcinoma: evidence for an autocrine tole in tumour growth. *Cancer Res* **59**: 720–727

Clozel JP and Clozel M (1989) Effects of endothelin on the coronary vascular bed in open-chest dogs. *Circ Res* **65**: 1193–1200

Clozel M, Gray GA, Breu V, Loffler BM and Osterwalder R (1992) The endothelin $ET_B$ receptor mediates both vasodilation and vasoconstriction *in vivo*. *Biochem Biophys Res Commun* **186**: 867–873

Eberl LP, Valdenaire O, Saintgiorgio V, Jeannin J-F and Juillerat-Jeanneret L (2000) Endothelin receptor blockade potentiates FasL-induced apoptosis in rat colon carcinoma cells. *Int J Cancer* **86**: 182–187

Hanahan D and Folkman J (1996) Patterns and emerging mechanisms of the angiogenic switch during tumorigenesis. *Cell* **86**: 353–364

Hirata Y, Takagi Y, Fukuda Y and Marumi F (1989) Endothelin is a potent mitogen for rat vascular smooth muscle cells. *Atherosclerosis* **78s**: 225–228

Inigagi H, Bishop AE, Eimoto T and Polak JM (1992) Autoradiographic localization of ET-1 binding sites in human colonic cancer tissue. *J Pathol* **168**: 263–267

Kitigawa KN, Tsutsumi K, Niwa M, Yamaga S, Anda T and Khalid H (1994) A selective $ET_A$ antagonist, BQ123, inhibits $^{125}$I-ET-1 binding of human meningiomas and antagonizes ET-1 induced proliferation. *Cell Mol Neurobiol* **14**: 105–118

Kojima K and Nihei Z (1995) Expression of ET-1 immunoreactivity in breast cancer. *Surg Oncol* **4**: 309–315

Kusuhara M, Yamaguchi K, Ohnishi A, Abe K, Kimura S, Oono H, Hori S and Nakamura Y (1989) Endothelin potentiates growth factor stimulated DNA synthesis in Swiss 3T3 cells. *Japn J Cancer Res* **80**: 302–305

Liu G, Henry KH Jr, Szczepankiewicz BG, Winn M, Kozmina NS, Boyd SA, Wasicak J, von Geldern TW, Wu-Wong JR, Chiou WJ, Dixon DG, Nguyen B, Marsh KC and Opgenorth TJ (1998) Pyrrolidine-3-carboxylic acids as endothelin antagonists. 3. Discovery of a potent 2-nonaryl, highly selective $ET_A$ antagonist (A-216546). *J Med Chem* **41**: 3261–3275

Loesch A, Turmaine M, Loizidou M, Crowe R, Ashraf S, Taylor I and Burnstock G (1997) Increase in immunoreactivity for ET-1 in blood vessels of rat liver metastases: experimental sarcoma and carcinoma. *J Anat* **191**: 291–299

Loizidou MC, Lawrance RJ, Holt S, Carty NJ, Cooper AJ, Alexander P and Taylor I (1991) Facilitation by partial hepatectomy of tumour growth within the rat liver following intraportal injection of syngeneic tumour cells. *Clin Exp Metastasis* **9**: 335–349

Luttrell LM, Daaka Y and Lefkowitz J (1999) Regulation of tyrosine kinase cascades by G protein coupled receptors. *Curr Opin Cell Biol* **11**: 177–183

Moraitis S, Langdon SP and Miller WR (1997) Endothelin expression and responsiveness in human ovarian cancer cell lines. *Eur J Cancer* **33**: 661–668

Nakamuta M, Ohashi M, Tabata S, Tanabe Y, Goto K, Naruse M, Naruse K, Hiroshige K and Nawata H (1993) High plasma concentrations of ET-like immunoreactivities in patients with hepatocellular carcinoma. *Am J Gastroenterol* **88**: 248–252

Nelson JB, Hedican SP, George DJ, Reddi AH, Piantadosi S, Eisenberger MA and Simon JW (1995) Identification of ET-1 in the pathophysiology of metastatic adenocarcinoma of the prostate. *Nat Med* **1**: 944–949

Nelson JB, Chan-Tack K, Hedican SP, Magnuson SR, Opgenorth TJ, Bova GS and Simons JW (1996) Endothelin-1 production and decreased ETB receptor expression in advanced prostatic cancer. *Cancer Res* **56**: 663–668

Oikawa T, Kushuhara M, Ishikawa S, Hitomi J, Kono A, Iwanaga T and Yamaguchi K (1994) Production of ET-1 and thrombomodulin by human pancreatic cancer cells. *Br J Cancer* **69**: 1059–1064

Ong A, Jowett T, Scoble J, O'Shea P, Varghese Z and Moorhead J (1993) Effect of cyclosporin A on endothelin synthesis by cultured human renal cortical epithelial cells. *Nephrol Dial Transplant* **8**: 748–753

Shankar A, Loizidou M and Taylor I (1996) The vascularity of colorectal liver metastases. *Eur J Surg Oncol* **22**: 389–396

Shankar A, Loizidou M, Aliev G, Fredericks S, Holt D, Boulos PB, Burnstock B and Taylor I (1998) Raised ET-1 levels in patients with colorectal liver metastases. *Br J Surg* **85**: 502–506

Shichiri M, Hirata Y, Nakajima T, Ando K, Imai T, Yanagisawa M, Masaki T and Marumo F (1991) Endothelin-1 is an autocrine/paracrine growth factor for human cancer cell lines. *J Clin Invest* **87**: 1867–1871

Shichiri M, Marumo F and Yukio H (1998) Endothelin-B receptor-mediated supression of endothelial apoptosis. *J Cardiovasc Pharmacol* **31**: S138–S141

Simonson MS, Wamm S, Mene P, Dubyak GR, Kester M, Nakazato Y, Sedor JR and Dunn MJ (1989) Endothelin stimulated phospholipase C, $Na^+/H^+$ exchange c-fos expression and mitogenesis in rat mesanglial cells. *J Clin Invest* **83**: 708–712

Simpson RA, Dickinson T, Porter KE, London NJM and Hemingway DM (2000) Raised levels of plasma Big ET-1 in patients with colorectal cancer. *Br J Surg* **87**: 1409–1413

Sutanto-Ward E, Sigurdson ER, Tremiterra S, Lincer R, Chapman D and Niedzwiecki D (1992) Adjuvant chemotherapy for colorectal hepatic metastases: role of route of administration and timing. *Surg Oncol* **1**: 87–95

Wilson J, Hunt SJ, Tang E, Wright N, Kelly E, Palmer S, Heys C, Mellor S, James R and Bialecki R (1999) Pharmacological profile of 2D1611, an orally active $ET_A$ antagonist. *J Pharmacol Exp Ther* **290**: 1085–1091

Yanagisawa M, Kurihara H, Kimura S, Tomobe Y, Kobayashi M, Mitsui Y, Yazaki Y, Goto K and Masaki T (1988) A novel potent vasoconstrictor peptide produced by vascular endothelial cells. *Nature* **332**: 411–415

*© 2001 Cancer Research Campaign*
